# Do additional nomograms based on the SEER dataset truly enhance survival prediction for patients with penile cancer?

**DOI:** 10.1002/cam4.70091

**Published:** 2024-08-09

**Authors:** Matthias May, Anton Kravchuk, Maarten Albersen, Ingmar Wolff

**Affiliations:** ^1^ Department of Urology St. Elisabeth Hospital Straubing, Brothers of Mercy Hospital Straubing Germany; ^2^ Caritas St. Josef Medical Center Teaching Hospital of the University Clinic Regensburg Regensburg Germany; ^3^ Department of Urology University Hospitals Leuven Leuven Belgium; ^4^ Department of Urology University Medicine Greifswald Greifswald Germany

With great interest, we read the recent publication by Luo et al., in which the authors focused on 3498 patients with penile cancer (PeCa) within the Surveillance, Epidemiology, and End Results (SEER) database.^1^ Their objective was to identify predictors for overall mortality (OM). For the construction of the nomogram, the study group was divided into a training and validation cohort in a 7‐to‐3 ratio, demonstrating overall stable calibrations and adequate predictive qualities (the *c*‐indices in the training and validation sets were approximately 0.74 each). Components of the final nomogram included the TNM stage, patient's age, race, marital status, histological subtype, tumor grade, and type of surgical intervention performed. An external validation of the nomogram was subsequently conducted using data from 103 of their own PeCa patients, which also showed good calibration and a *c*‐index of 0.78.

In addition to the missing information on median follow‐up, there are several comments on the manuscript, and we would be interested in the authors' perspectives on these points: (1) The information provided in the abstract differs significantly from that given in the manuscript text (3154 vs. 3498 PeCa patients, 2:1 vs. 7:3 ratio). (2) Information on the validity of the N‐status is missing (clinical or pathological)—an Nx proportion of 11% likely excludes a pathological assessment of the N‐status, as it is hardly conceivable that nearly 9 out of 10 patients underwent an inguinal lymph node dissection (ILND). By the way, can data on the prognostic value of an ILND be derived from the dataset? (3) Mx shows a relative 40% lower OM compared to M0, which allows inferences about the validity of the data. (4) The distinction between verrucous carcinoma and squamous cell carcinoma in the histological classification seems unclear, as the former is merely a subtype of the latter. (5) The lack of prognostic differences between tumor stages Ta, T1, and T2 renders the nomogram unreliable from our perspective; similarly, the prognostic difference between T3 and Ta is approximately equivalent to that of patients being unmarried. (6) For PeCa grade 4, a 95% confidence interval of 0.72–2.26 was reported compared to unknown grade, which cannot correspond to a *p*‐value of <0.001 (multivariate Cox model^1^).

Additionally, it is remarkable how many PeCa survival analyses based on the SEER database continue to be published—no less than eight original studies since November 2023 (see Supporting Information). For this reason, we performed a bibliometric analysis of all 60 original studies listed in PubMed involving survival analyses of PeCa patients based on the SEER database (often coupled with nomogram creation) (Figure 1). For the first time, the analysis presented the connections between the authors, their institutions, and countries, as well as the target journals that publish survival analyses of PeCa patients based on the SEER database.

**FIGURE 1:**
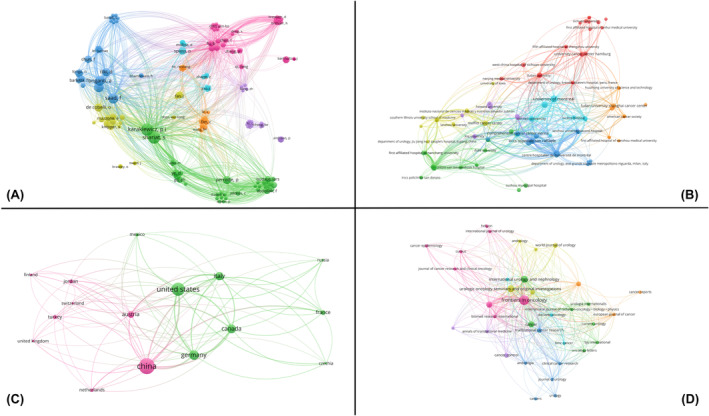
Bibliometric analyses were conducted based on a PubMed search focused on original articles concerning patients with penile cancer and survival analyses using the Surveillance, Epidemiology, and End Results (SEER) database. The studies included were those published up to the e‐publication date of June 10, 2024. The bibliometric analyses encompassed the names (A), institutions (B), and countries (C) of the authors, as well as the journals (D) of the publications.

Overall, we are very uncertain whether more of these studies based on SEER data are truly necessary. Instead, nomograms for survival prediction based on specific and well‐captured histopathological criteria and the integration of meaningful biomarkers (molecular taxonomy) of immune, stromal, and tumor microenvironment are needed. Additionally, we believe that the international research community should focus on identifying better predictive rather than purely prognostic criteria for specific therapies to ultimately improve long‐term prognosis of PeCa patients.

## AUTHOR CONTRIBUTIONS

**Matthias May:** Conceptualization (lead); data curation (lead); formal analysis (lead); investigation (lead); methodology (lead); project administration (lead); software (lead); supervision (lead); writing – original draft (lead); writing – review and editing (lead). **Anton Kravchuk:** Conceptualization (supporting); data curation (supporting); formal analysis (supporting); software (equal). **Maarten Albersen:** Conceptualization (supporting); supervision (supporting); writing – review and editing (equal). **Ingmar Wolff:** Project administration (supporting); supervision (supporting); writing – review and editing (supporting).

## CONFLICT OF INTEREST STATEMENT

The authors have no conflict of interest to declare.

## Supporting information


Data S1.

## Data Availability

The submitted manuscript is a ‘Letter’, hence a ‘Data Availability Statement’ is generally uncommon. Nevertheless, the ‘Letter’ includes a bibliometric analysis, with all the search terms used for identifying the considered research works provided in the Supporting Information. The linkage of the identified research works during the bibliometric analysis can be made available upon request.
