# Supplementary material for: Do additional nomograms based on the SEER dataset truly enhance survival prediction for patients with penile cancer?
Source: Cancer Med. 2024 Aug 9;13(15):e70091. doi: 10.1002/cam4.70091 (PMC11310760; doi:10.1002/cam4.70091)
Supplement: Supplementary file 1 — Data S1. [file CAM4-13-e70091-s001.docx]

**Search Terms:**

(SEER OR Surveillance, Epidemiology, and End Results) AND (penile) AND (survival)

**Period of PubMed search:** From the inception of PubMed records to June 10, 2024

**Hits:** 105 papers

**Research papers suitable based on title and abstract evaluation:** 60 papers

**List of research papers referred to in the letter to the editor:**

1: Liu S, Shen Z, Yang H, Wang J, Wang X, Gong Y, Liu S, Lu Z, Huang T.

Development and validation of HPV-associated and HPV-independent penile squamous

cell carcinoma prognostic nomogram. Int Urol Nephrol. 2024 Apr 29. doi:

10.1007/s11255-024-04043-3. Epub ahead of print. PMID: 38679654.

2: Luo J, Hu J, Mulati Y, Wu Z, Lai C, Kong D, Liu C, Xu K. Developing and

validating a nomogram for penile cancer survival: A comprehensive study based on

SEER and Chinese data. Cancer Med. 2024 Apr;13(7):e7111. doi: 10.1002/cam4.7111.

PMID: 38566587; PMCID: PMC10988236.

3: Chen D, Liang S, Chen J, Li K, Mi H. Machine learning-based overall and

cancer-specific survival prediction of M0 penile squamous cell carcinoma：A

population-based retrospective study. Heliyon. 2023 Dec 8;10(1):e23442. doi:

10.1016/j.heliyon.2023.e23442. PMID: 38163093; PMCID: PMC10755306.

4: Ashley LW, Sutton KF, Ju A, Edwards G, Pasli M, Bhatt A. A SEER database

retrospective cohort of 547 patients with penile non-squamous cell carcinoma:

demographics, clinical characteristics, and outcomes. Front Oncol. 2023 Oct

31;13:1271913. doi: 10.3389/fonc.2023.1271913. PMID: 38023122; PMCID:

PMC10644775.

5: Scheipner L, Tappero S, Piccinelli ML, Barletta F, Garcia CC, Incesu RB,

Morra S, Tian Z, Saad F, Shariat SF, Terrone C, De Cobelli O, Briganti A, Chun

FKH, Tilki D, Longo N, Seles M, Ahyai S, Karakiewicz PI. Differences in overall

survival of penile cancer patients versus population-based controls. Int J Urol.

2024 Mar;31(3):274-279. doi: 10.1111/iju.15346. Epub 2023 Nov 28. PMID:

38014575.

6: Li C, Liu D, Yao K, Liu J, Wang J, Zhang Y, Cui L, Wang L. Could the New pN

Staging Classification Better Predict the Prognosis of Penile Cancer? A

Population-Based Analysis. Arch Esp Urol. 2023 Sep;76(7):481-486. doi:

10.56434/j.arch.esp.urol.20237607.59. PMID: 37867332.

7: Tang CP, Zhou K, Sheng ZC, Zhou YL, Zhou SG, Xu S. [Clinical characteristics

and prognostic factors of distant metastatic penile cancer]. Zhonghua Nan Ke

Xue. 2023 Feb;29(2):151-157. Chinese. PMID: 37847087.

8: Yang S, Chang W, Zhang B, Hou Q, Zhang B, Kang Y, Yin Y, Wan J, Shang P.

Development and validation of a predictive model for penile cancer based on the

surveillance, epidemiology, and end results database and multi-center cases. J

Cancer Res Clin Oncol. 2023 Nov;149(15):13665-13676. doi:

10.1007/s00432-023-04784-1. Epub 2023 Jul 31. PMID: 37522926.

9: Scheipner L, Cano Garcia C, Barletta F, Incesu RB, Morra S, Baudo A, Assad A,

Tian Z, Saad F, Shariat SF, Chun FKH, Briganti A, Tilki D, Longo N, Carmignani

L, Leitsmann M, Ahyai S, Karakiewicz PI. Regional differences in penile cancer

patient characteristics and treatment rates across the United States. Cancer

Epidemiol. 2023 Oct;86:102424. doi: 10.1016/j.canep.2023.102424. Epub 2023 Jul

26. PMID: 37506474.

10: Akinyemi OA, Fasokun ME, Weldeslase TA, Adeoye O, Coleman PW. Influence of

Race/Ethnicity and Household Median Income on Penile Cancer Mortality. Cureus.

2023 Jun 24;15(6):e40909. doi: 10.7759/cureus.40909. PMID: 37496530; PMCID:

PMC10366004.

11: Candelario NC, Molina E, Bourlon MT, Kim SP, Kessler ER, Spiess PE, Flaig

TW. Racial differences in survival for early stage (T1) penile cancer: Analysis

from the SEER database. Urol Oncol. 2023 Aug;41(8):359.e15-359.e23. doi:

10.1016/j.urolonc.2023.05.018. Epub 2023 Jun 20. PMID: 37344326; PMCID:

PMC10658609.

12: Xie Z, Zhan X, Zheng Y, Liu Y, Chen T, Jiang M, Li Y, Fu B. High

cardiovascular disease mortality after penile squamous cell carcinomas

diagnosis: Results from the United States SEER population, 2005-2016. Front

Oncol. 2022 Oct 14;12:1004791. doi: 10.3389/fonc.2022.1004791. PMID: 36313644;

PMCID: PMC9615145.

13: Song P, Wu X, Yang L, Ma K, Liu Z, Zhou J, Chen J, Zhu Q, Dong Q. Second

Malignant Tumors and Non-Tumor Causes of Death for Patients With Penile Cancer

During Their Survivorship. Cancer Control. 2022 Jan-Dec;29:10732748221134789.

doi: 10.1177/10732748221134789. PMID: 36267038; PMCID: PMC9597479.

14: Tappero S, Piccinelli M, Barletta F, Panunzio A, Cano Garcia C, Incesu RB,

Tian Z, Parodi S, Dell'Oglio P, De Cobelli O, Briganti A, Antonelli A, Chun FKH,

Graefen M, Saad F, Shariat SF, Suardi NR, Borghesi M, Terrone C, Karakiewicz PI.

Effect of inguinal lymph node dissection in lymph node negative patients with

squamous cell carcinoma of the penis. World J Urol. 2023 Jan;41(1):119-125. doi:

10.1007/s00345-022-04184-z. Epub 2022 Oct 14. PMID: 36239810.

15: Li K, Le X, Wang J, Fan C, Sun J. Tumor Location May Independently Predict

Survival in Patients With M0 Squamous Cell Carcinoma of the Penis. Front Oncol.

2022 Jul 5;12:927088. doi: 10.3389/fonc.2022.927088. PMID: 35865480; PMCID:

PMC9294313.

16: Bourlon MT, Verduzco-Aguirre H, Molina E, Meyer E, Kessler E, Kim SP, Spiess

PE, Flaig T. Patterns of Treatment and Outcomes in Older Men With Penile Cancer:

A SEER Dataset Analysis. Front Oncol. 2022 Jun 29;12:926692. doi:

10.3389/fonc.2022.926692. PMID: 35847850; PMCID: PMC9277543.

17: Eminaga O, Shkolyar E, Breil B, Semjonow A, Boegemann M, Xing L, Tinay I,

Liao JC. Artificial Intelligence-Based Prognostic Model for Urologic Cancers: A

SEER-Based Study. Cancers (Basel). 2022 Jun 26;14(13):3135. doi:

10.3390/cancers14133135. PMID: 35804904; PMCID: PMC9264864.

18: Deng X, Liu Y, Zhan X, Chen T, Jiang M, Jiang X, Chen L, Fu B. Trends in

Incidence, Mortality, and Survival of Penile Cancer in the United States: A

Population-Based Study. Front Oncol. 2022 Jun 17;12:891623. doi:

10.3389/fonc.2022.891623. PMID: 35785206; PMCID: PMC9248743.

19: Li R, Cheng K, Wei Z, Liu Z, Peng X. The Development and Validation of a

Nomogram Incorporating Clinical, Pathological, and Therapeutic Features to

Predict Overall Survival in Patients With Penile Cancer: A SEER-Based Study.

Front Oncol. 2022 Apr 5;12:840367. doi: 10.3389/fonc.2022.840367. PMID:

35449579; PMCID: PMC9016192.

20: Chen YB, Liu YW, Gao L, Tang LY, Guo J, Tian YC, You PH. Development and

Verification of Prognostic Nomogram for Penile Cancer Based on the SEER

Database. Biomed Res Int. 2022 Apr 4;2022:8752388. doi: 10.1155/2022/8752388.

PMID: 35419456; PMCID: PMC9001101.

21: Zhan X, Chen L, Jiang M, Fu B. Get insight into the cause of death

distribution and epidemiology of penile squamous cell carcinoma: A population-

based study. Cancer Med. 2022 Jun;11(11):2308-2319. doi: 10.1002/cam4.4614. Epub

2022 Mar 3. PMID: 35238488; PMCID: PMC9160805.

22: Li K, Wu G, Fan C, Yuan H. The prognostic significance of primary tumor size

in squamous cell carcinoma of the penis. Discov Oncol. 2021 Jul 19;12(1):22.

doi: 10.1007/s12672-021-00416-7. PMID: 35201454; PMCID: PMC8777549.

23: Osazuwa-Peters N, Simpson MC, Rohde RL, Challapalli SD, Massa ST, Adjei

Boakye E. Differences in Sociodemographic Correlates of Human Papillomavirus-

Associated Cancer Survival in the United States. Cancer Control. 2021 Jan-

Dec;28:10732748211041894. doi: 10.1177/10732748211041894. PMID: 34696619; PMCID:

PMC8552385.

24: Li H, Ma Y, Jian Z, Jin X, Xiang L, Li H, Wang K. Lymph Node Dissections for

T3T4 Stage Penile Cancer Patients Without Preoperatively Detectable Lymph Node

Metastasis Bring More Survival Benefits: A Propensity Matching Analysis. Front

Oncol. 2021 Sep 24;11:712553. doi: 10.3389/fonc.2021.712553. PMID: 34631537;

PMCID: PMC8497980.

25: Yu J, Long Q, Zhang Z, Liao S, Zheng F. The prognostic value of lymph node

ratio in comparison to positive lymph node count in penile squamous cell

carcinoma. Int Urol Nephrol. 2021 Dec;53(12):2527-2540. doi:

10.1007/s11255-021-02996-3. Epub 2021 Sep 28. PMID: 34585313; PMCID: PMC8599252.

26: Wenzel M, Siron N, Collà Ruvolo C, Nocera L, Würnschimmel C, Tian Z, Shariat

SF, Saad F, Briganti A, Tilki D, Banek S, Kluth LA, Roos FC, Chun FKH,

Karakiewicz PI. Temporal trends, tumor characteristics and stage-specific

survival in penile non-squamous cell carcinoma vs. squamous cell carcinoma.

Cancer Causes Control. 2022 Jan;33(1):25-35. doi: 10.1007/s10552-021-01493-3.

Epub 2021 Sep 2. PMID: 34476653; PMCID: PMC8738356.

27: Gao P, Zhu T, Gao J, Li H, Liu X, Zhang X. Impact of Examined Lymph Node

Count and Lymph Node Density on Overall Survival of Penile Cancer. Front Oncol.

2021 Jul 7;11:706531. doi: 10.3389/fonc.2021.706531. PMID: 34307174; PMCID:

PMC8293298.

28: Hensley PJ, Loomis J, Bylund JR, James AC. Demographic disparities of penile

cancer in Appalachian Kentucky. Can J Urol. 2021 Jun;28(3):10713-10718. PMID:

34129468.

29: Chen WK, Wu ZG. Adding radiotherapy based on chemotherapy can improve

cancer-specific survival in N3 penile cancer: a SEER-based study. Transl Androl

Urol. 2020 Dec;9(6):2587-2595. doi: 10.21037/tau-20-1044. PMID: 33457231; PMCID:

PMC7807347.

30: Qi F, Wei X, Zheng Y, Ren X, Li X, Zhao E. Incidence trends and survival

outcomes of penile squamous cell carcinoma: evidence from the Surveillance,

Epidemiology and End Results population-based data. Ann Transl Med. 2020

Nov;8(21):1428. doi: 10.21037/atm-20-1802. PMID: 33313173; PMCID: PMC7723588.

31: Bhambhvani HP, Greenberg DR, Parham MJ, Eisenberg ML. A population-level

analysis of nonsquamous penile cancer: The importance of histology. Urol Oncol.

2021 Feb;39(2):136.e1-136.e10. doi: 10.1016/j.urolonc.2020.11.025. Epub 2020 Nov

27. PMID: 33257222.

32: Bhattacharjee A, Roy G, Budukh A, Dikshit R, Patil VM, Joshi A, Noronha V,

Prabash K, Roy P. A competing risk analysis of death patterns in male

genitourinary cancer. Cancer Rep (Hoboken). 2020 Aug;3(4):e1174. doi:

10.1002/cnr2.1174. Epub 2019 Apr 4. PMID: 32794635; PMCID: PMC7941467.

33: Liu W, Luo Y, Wang G, Li N, Wang Z, Lei J, Wang X. Conditional survival

after surgery for patients with penile cancer. Andrology. 2020

Nov;8(6):1744-1752. doi: 10.1111/andr.12856. Epub 2020 Jul 12. PMID: 32619060.

34: Zheng W, Li K, Zhu W, Ding Y, Wu Q, Tang Q, Lu C, Zhao Q, Yu S, Guo C.

Nomogram prediction of overall survival based on log odds of positive lymph

nodes for patients with penile squamous cell carcinoma. Cancer Med. 2020

Aug;9(15):5425-5435. doi: 10.1002/cam4.3232. Epub 2020 Jun 10. PMID: 32519819;

PMCID: PMC7402844.

35: Huang T, Xu Y, Hu G, Zhu D, Xiao S, He R. Racial/ethnic disparities in

penile squamous cell carcinoma incidences, clinical characteristics, and

outcomes: A population-based study, 2004-2016. Urol Oncol. 2020

Aug;38(8):688.e11-688.e19. doi: 10.1016/j.urolonc.2020.03.003. Epub 2020 Apr 25.

PMID: 32340796.

36: Xu W, Qi F, Liu Y, Zheng L, Kang Z. Nomograms to predict overall and cancer-

specific survival in patients with penile cancer. Transl Cancer Res. 2020

Apr;9(4):2326-2339. doi: 10.21037/tcr.2020.03.77. PMID: 35117593; PMCID:

PMC8798233.

37: Mistretta FA, Cyr SJ, Palumbo C, Mazzone E, Knipper S, Tian Z, Nazzani S,

Montanari E, Tilki D, Briganti A, Shariat SF, Perrotte P, Saad F, de Cobelli O,

Karakiewicz PI. Adherence to Guideline Recommendations for Perioperative

Chemotherapy in Patients with pN2-3 M0 Squamous Cell Carcinoma of the Penis:

Temporal Trends and Survival Outcomes. Clin Oncol (R Coll Radiol). 2020

Apr;32(4):e93-e101. doi: 10.1016/j.clon.2019.10.001. Epub 2019 Nov 7. PMID:

31706712.

38: Zheng QL, Wu YP, Zhang ZP, Xu N. Partial penectomy or total penectomy for T1

and T2 squamous cell carcinoma of the penis? Transl Cancer Res. 2019

Sep;8(5):1750-1755. doi: 10.21037/tcr.2019.08.37. PMID: 35116925; PMCID:

PMC8798828.

39: Khalil MI, Wan F, Eltahawy E, Davis R, Spiess PE, Bissada NK, Kamel MH.

Survival Following Salvage Surgery after Failed Radiotherapy for Penile Cancer:

A SEER-Based Study. Curr Urol. 2019 May 10;12(3):142-146. doi:

10.1159/000489432. PMID: 31316322; PMCID: PMC6613318.

40: Mistretta FA, Palumbo C, Knipper S, Mazzone E, Pecoraro A, Tian Z, Musi G,

Perrotte P, Montanari E, Shariat SF, Saad F, Briganti A, de Cobelli O,

Karakiewicz PI. Conditional survival of patients with stage I-III squamous cell

carcinoma of the penis: temporal changes in cancer-specific mortality. World J

Urol. 2020 Mar;38(3):725-732. doi: 10.1007/s00345-019-02869-6. Epub 2019 Jul 11.

PMID: 31297629.

41: Mistretta FA, Mazzone E, Palumbo C, Knipper S, Tian Z, Nazzani S, Tilki D,

Musi G, Perrotte P, Montanari E, Shariat SF, Saad F, Briganti A, de Cobelli O,

Karakiewicz PI. Adherence to guideline recommendations for lymph node dissection

in squamous cell carcinoma of the penis: Effect on survival and complication

rates. Urol Oncol. 2019 Sep;37(9):578.e11-578.e19. doi:

10.1016/j.urolonc.2019.05.024. Epub 2019 Jul 8. PMID: 31296420.

42: Mao W, Zhang Z, Huang X, Fan J, Geng J. Marital Status and Survival in

Patients with Penile Cancer. J Cancer. 2019 Jun 2;10(12):2661-2669. doi:

10.7150/jca.32037. PMID: 31258774; PMCID: PMC6584924.

43: Li K, Sun J, Wei X, Wu G, Wang F, Fan C, Yuan H. Prognostic value of

lymphovascular invasion in patients with squamous cell carcinoma of the penis

following surgery. BMC Cancer. 2019 May 21;19(1):476. doi:

10.1186/s12885-019-5714-1. PMID: 31113402; PMCID: PMC6528249.

44: Mao W, Huang X, Kong M, Fan J, Geng J. More lymph node dissection improves

survival in patients with newly diagnosed lymph node-positive penile cancer. Int

Urol Nephrol. 2019 Apr;51(4):641-654. doi: 10.1007/s11255-019-02084-7. Epub 2019

Feb 13. PMID: 30758771.

45: Zhu Y, Gu WJ, Xiao WJ, Wang BH, Azizi M, Spiess PE, Ye DW. Important

Therapeutic Considerations in T1b Penile Cancer: Prognostic Significance and

Adherence to Treatment Guidelines. Ann Surg Oncol. 2019 Feb;26(2):685-691. doi:

10.1245/s10434-018-7066-5. Epub 2018 Dec 18. PMID: 30565040.

46: Wei Z, Yu Z, Li H, Peng W, Zhang J, Zhang Y, Song W, Liu J, Yang W, Wang T.

The appropriate number of negative lymph nodes dissection for nonmetastatic

penile cancer. Andrologia. 2019 Feb;51(1):e13154. doi: 10.1111/and.13154. Epub

2018 Sep 25. PMID: 30255596.

47: Romanelli M, Issa T, Zahnd W, Dynda D, Alanee S. Impact of County Rurality

and Urologist Density on the Practice of Inguinal Lymph Node Dissection and

Mortality in Patients Diagnosed with Squamous Cell Carcinoma of the Penis. Ann

Surg Oncol. 2018 Jan;25(1):334-341. doi: 10.1245/s10434-017-6211-x. Epub 2017

Nov 6. PMID: 29110272.

48: Zhu Y, Gu WJ, Wang HK, Gu CY, Ye DW. Surgical treatment of primary disease

for penile squamous cell carcinoma: A Surveillance, Epidemiology, and End

Results database analysis. Oncol Lett. 2015 Jul;10(1):85-92. doi:

10.3892/ol.2015.3221. Epub 2015 May 18. PMID: 26170981; PMCID: PMC4487029.

49: Zhu Y, Gu CY, Ye DW. Population-based assessment of the number of lymph

nodes removed in the treatment of penile squamous cell carcinoma. Urol Int.

2014;92(2):186-93. doi: 10.1159/000354401. Epub 2013 Nov 12. PMID: 24246932.

50: Burt LM, Shrieve DC, Tward JD. Stage presentation, care patterns, and

treatment outcomes for squamous cell carcinoma of the penis. Int J Radiat Oncol

Biol Phys. 2014 Jan 1;88(1):94-100. doi: 10.1016/j.ijrobp.2013.08.013. Epub 2013

Oct 9. PMID: 24119832.

51: Zhu Y, Gu CY, Ye DW. Validation of the prognostic value of lymph node ratio

in patients with penile squamous cell carcinoma: a population-based study. Int

Urol Nephrol. 2013 Oct;45(5):1263-71. doi: 10.1007/s11255-013-0502-3. Epub 2013

Jul 23. PMID: 23877663.

52: Verhoeven RH, Janssen-Heijnen ML, Saum KU, Zanetti R, Caldarella A,

Holleczek B, Brewster DH, Hakulinen T, Horenblas S, Brenner H, Gondos A; EUNICE

Survival Working Group. Population-based survival of penile cancer patients in

Europe and the United States of America: no improvement since 1990. Eur J

Cancer. 2013 Apr;49(6):1414-21. doi: 10.1016/j.ejca.2012.10.029. Epub 2012 Dec

8. PMID: 23231984.

53: Thuret R, Sun M, Budaus L, Abdollah F, Liberman D, Shariat SF, Iborra F,

Guiter J, Patard JJ, Perrotte P, Karakiewicz PI. A population-based analysis of

the effect of marital status on overall and cancer-specific mortality in

patients with squamous cell carcinoma of the penis. Cancer Causes Control. 2013

Jan;24(1):71-9. doi: 10.1007/s10552-012-0091-y. Epub 2012 Oct 30. PMID:

23109172.

54: Thuret R, Sun M, Abdollah F, Budaus L, Shariat SF, Iborra F, Guiter J,

Patard JJ, Karakiewicz PI. Competing-risks analysis in patients with T1 squamous

cell carcinoma of the penis. BJU Int. 2013 Apr;111(4 Pt B):E174-9. doi:

10.1111/j.1464-410X.2012.11505.x. Epub 2012 Oct 12. PMID: 23057865.

55: Tyson MD, Etzioni DA, Wisenbaugh ES, Andrews PE, Humphreys MR, Ferrigni RG,

Swanson SK, Castle EP. Anatomic site-specific disparities in survival outcomes

for penile squamous cell carcinoma. Urology. 2012 Apr;79(4):804-8. doi:

10.1016/j.urology.2011.12.047. Epub 2012 Mar 3. PMID: 22381248.

56: Thuret R, Sun M, Abdollah F, Budaus L, Lughezzani G, Liberman D, Morgan M,

Johal R, Jeldres C, Latour M, Shariat SF, Iborra F, Guiter J, Patard JJ,

Perrotte P, Karakiewicz PI. Tumor grade improves the prognostic ability of

American Joint Committee on Cancer stage in patients with penile carcinoma. J

Urol. 2011 Feb;185(2):501-7. doi: 10.1016/j.juro.2010.09.111. Epub 2010 Dec 17.

PMID: 21167526.

57: Johnson TV, Hsiao W, Delman KA, Jani AB, Brawley OW, Master VA. Extensive

inguinal lymphadenectomy improves overall 5-year survival in penile cancer

patients: results from the Surveillance, Epidemiology, and End Results program.

Cancer. 2010 Jun 15;116(12):2960-6. doi: 10.1002/cncr.25091. PMID: 20564401.

58: Zini L, Cloutier V, Isbarn H, Perrotte P, Capitanio U, Jeldres C, Shariat

SF, Saad F, Arjane P, Duclos A, Lattouf JB, Montorsi F, Karakiewicz PI. A simple

and accurate model for prediction of cancer-specific mortality in patients

treated with surgery for primary penile squamous cell carcinoma. Clin Cancer

Res. 2009 Feb 1;15(3):1013-8. doi: 10.1158/1078-0432.CCR-08-1888. PMID:

19188173.

59: Hernandez BY, Barnholtz-Sloan J, German RR, Giuliano A, Goodman MT, King JB,

Negoita S, Villalon-Gomez JM. Burden of invasive squamous cell carcinoma of the

penis in the United States, 1998-2003. Cancer. 2008 Nov 15;113(10

Suppl):2883-91. doi: 10.1002/cncr.23743. PMID: 18980292; PMCID: PMC2693711.

60: Rippentrop JM, Joslyn SA, Konety BR. Squamous cell carcinoma of the penis:

evaluation of data from the surveillance, epidemiology, and end results program.

Cancer. 2004 Sep 15;101(6):1357-63. doi: 10.1002/cncr.20519. PMID: 15316902.
